# Identical sets of methylated and nonmethylated genes in *Ciona intestinalis* sperm and muscle cells

**DOI:** 10.1186/1756-8935-6-38

**Published:** 2013-11-11

**Authors:** Miho M Suzuki, Akiko Yoshinari, Madoka Obara, Shohei Takuno, Shuji Shigenobu, Yasunori Sasakura, Alastair RW Kerr, Shaun Webb, Adrian Bird, Atsuo Nakayama

**Affiliations:** 1 Department of Embryology, Institute for Developmental Research, Aichi Human Service Center, 713-8 Kamiya-Cho, Kasugai, Aichi 480-0392, Japan; 2 Japan Science and Technology Agency, PRESTO, 4-1-8 Honcho, Kawaguchi, Saitama 332-0012, Japan; 3 National Institute for Basic Biology, 38 Nishigonaka, Myodaiji, Okazaki, Aichi 444-8585, Japan; 4 Graduate University for Advanced Studies, Hayama, Kanagawa 240-0193, Japan; 5 Shimoda Marine Research Center, University of Tsukuba, 5-10-1 Shimoda, Shizuoka 415-0025, Japan; 6 The Wellcome Trust Centre for Cell Biology, The University of Edinburgh, Michael Swann Building, The King’s Buildings, Edinburgh EH9 3JR, UK

## Abstract

**Background:**

The discovery of gene body methylation, which refers to DNA methylation within gene coding region, suggests an as yet unknown role of DNA methylation at actively transcribed genes. In invertebrates, gene bodies are the primary targets of DNA methylation, and only a subset of expressed genes is modified.

**Results:**

Here we investigate the tissue variability of both the global levels and distribution of 5-methylcytosine (5mC) in the sea squirt *Ciona intestinalis*. We find that global 5mC content of early developmental embryos is high, but is strikingly reduced in body wall tissues. We chose sperm and adult muscle cells, with high and reduced levels of global 5mC respectively, for genome-wide analysis of 5mC targets. By means of CXXC-affinity purification followed by deep sequencing (CAP-seq), and genome-wide bisulfite sequencing (BS-seq), we designated body-methylated and unmethylated genes in each tissue. Surprisingly, body-methylated and unmethylated gene groups are identical in the sperm and muscle cells. Our analysis of microarray expression data shows that gene body methylation is associated with broad expression throughout development. Moreover, transgenic analysis reveals contrasting gene body methylation at an identical gene-promoter combination when integrated at different genomic sites.

**Conclusions:**

We conclude that gene body methylation is not a direct regulator of tissue specific gene expression in *C. intestinalis*. Our findings reveal constant targeting of gene body methylation irrespective of cell type, and they emphasize a correlation between gene body methylation and ubiquitously expressed genes. Our transgenic experiments suggest that the promoter does not determine the methylation status of the associated gene body.

## Background

DNA methylation is an epigenetic modification that is widely employed in eukaryotes, including plants, fungi and animals, and serves multiple critical functions [1-3]. In eukaryotes, this chemical modification is deposited at the 5 position of cytosine by members of the *DNA methyltransferase* (*DNMT*) family of enzymes. 5-methylcytosine (5mC) is distributed nonrandomly in the genome. Recent approaches have enabled genomewide studies of 5mC distribution and demonstrated that the pattern changes during early mouse embryogenesis [4] and adult mouse stem cell differentiation [5], representing alteration of local epigenetic states in differentiating cells. It has been recognized that some of the changes occur at gene promoters and lead to stable gene silencing [6-8]. Recently, an unexpected target of DNA methylation has also been revealed. In several animal and plant genomes, the transcribed regions of genes, or “gene bodies”, including exons and introns, have higher levels of DNA methylation than neighboring sequences [9-12]. Typically, methylation occurs in the intragenic region and falls sharply at the 5′ and 3′ ends of the transcription unit. This characteristic modification pattern can be superimposed onto the regions occupied by elongating RNA polymerase II [10], raising the possibility that gene body methylation is associated with transcriptional elongation. In line with this hypothesis, gene body methylation often correlates with actively transcribed genes, but not with silent genes, in plants and animals, including human cells [9,12-15]. The role of gene body methylation, however, remains enigmatic [16].

Among invertebrates, gene bodies are the primary targets for DNA methylation. In our previous study, about 60% of all genes were deduced to be methylated in an invertebrate chordate, *Ciona intestinalis*[11]. Lately, genomewide bisulfite sequencing (BS-seq) of multiple invertebrates, including *C. intestinalis*, *Apis mellifera*, *Bombyx mori* and *Nematostella vectensis*, confirmed that prominent gene body methylation is a common feature in the invertebrate genomes [13,14,17-19]. Unlike promoter methylation, gene body methylation is not related simply to the gene expression level [9,10]. A subset of expressed genes is methylated, whereas unmethylated genes are also expressed at equivalent or even higher levels. Evidently, methylated genes are often evolutionarily conserved and encode essential cellular functions [11,20]. These genes tend to be expressed in a wide variety of tissues, but the mechanism by which a subgroup of expressed genes becomes methylated is not clear. Recent genomewide studies of invertebrate DNA methylation have used a single tissue or whole adult bodies [13,14,17,21,22]. Thus, dynamic changes of gene body methylation in somatic cells during development or at the tissue differentiation event remain unknown. Motivated to investigate this phenomenon, we decided to study tissue variability of gene body methylation in an invertebrate animal.

The pattern of gene body DNA methylation found in invertebrates is strikingly different from that of globally methylated mammalian genomes [23]. The genome of *C. intestinalis* provides an archetypal example of the gene body methylation pattern. BS-seq of selected regions of the sperm genome shows very high levels of DNA methylation in gene bodies (almost all CpG sites are methylated in 90% to 100% of the cells) [11], whereas promoter and intergenic regions are completely unmethylated. This “black or white” modification pattern is not biased toward transposons, which are often unmethylated in this animal [11,24]. Thus, the major targets of DNA methylation in the *C. intestinalis* genome are euchromatic regions. In the current study, we first investigated global 5mC content in early developmental stages and adult tissues to find the stages or tissues in which DNA methylation varies. Next, we chose sperm and adult muscle, which have high and low levels of global 5mC, respectively, for genomewide mapping of 5mC. Using CXXC affinity purification and deep sequencing (CAP-seq), together with analysis of published BS-seq data, we designated body-methylated and unmethylated genes in each tissue. The intertissue comparison of gene body methylation demonstrates that an identical set of genes is targeted for methylation in the sperm and muscle cells. This result suggests that gene body methylation is not a major regulator of tissue-specific expression in *C. intestinalis*. By analyzing microarray expression data, we show that gene body methylation is associated with broad expression throughout development. Additionally, we found that a gene body became methylated or remained unmethylated in two independent transgenic lines, despite possessing the same driving promoter. This finding suggests that the promoter is not the critical determinant of gene body methylation status.

## Results

### Global 5mC content of *Ciona intestinalis* genome is kept at high levels in early developmental stages and reduced in adult body wall tissues

**Figure 1:**
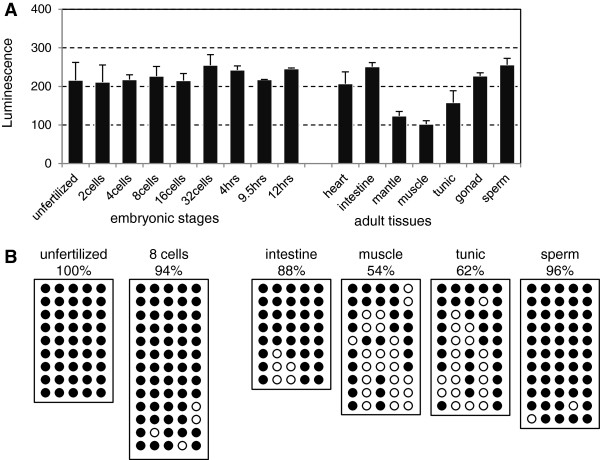
**DNA methylation in early developmental stages and adult tissues. (A)** Global DNA methylation level of genomic DNA from unfertilized eggs, embryonic stages and adult tissues were determined by an enzyme-linked immunosorbent assay specific for methylated DNA. Error bars indicate standard deviation (*n* = 3) **(B)** Bisulfite sequencing of *EF-1α* gene body region. Examined selected stages and tissues are noted above boxes. The sequencing results from eight to thirteen clones are shown in boxes. Each horizontal line represents the sequencing result of one subclone. Methylated CpG sites are shown as solid circles, and open circles indicate unmethylated CpG sites.

To measure global DNA methylation levels during development, 5mC content was determined via an enzyme-linked immunosorbent assay reaction using an anti-5mC antibody. Genomic DNA from 10 developmental stages were measured: unfertilized eggs, 2 cells, 4 cells, 8 cells, 16 cells, 32 cells, 64 cells, 4 hours after fertilization (early gastrula), 9.5 hours (early tailbud) and 12 hours (late tailbud). The results showed little change at different stages of development (Figure 1A). The *C. intestinalis* genome does not undergo global demethylation of parental genomes after fertilization, unlike in the early zygotes of mammals [4,25]. BS-seq showed that the detailed methylation status of *EF-1α* gene body region in unfertilized and eight cells stages (Figure 1B) was relatively constant at 100% and 94% of CpG sites, respectively.

In contrast to the constant levels of methylation in different stages of development, a large intertissue difference in global 5mC content was observed in some adult samples. Heart, intestine and reproductive tissues from gonad and mature sperm showed high 5mC content equivalent to early developmental stages (Figure 1A). Accordingly, at a single-gene level, the *EF-1α* gene body was highly methylated in both intestine and sperm (88% and 96%, respectively) (Figure 1B). On the other hand, levels of 5mC in body wall tissues, such as the cells from tunic (outermost layer of the body), outer mantle (thin epidermis and underlying connective tissue) and muscle, were approximately half those seen in embryos (Figure 1A). The reduced level of global 5mC in the body wall could be due to the locus-specific methylation changes, to an overall average decrease in DNA methylation levels, or to both. In favor of the second possibility, BS-seq of the *EF-1α* gene body in muscle and tunic cells was about half the level in embryos (54% and 62% of CpG sites, respectively) (Figure 1B). Loss of methylated CpG sites appeared to be random within this methylated domain, with a variable frequency at each CpG site. The global 5mC content was not correlated to the relative expression level of *DNMT*s in the adult tissues we examined (Additional file 1: Figure S1).

### Stable targeting of gene body methylation in sperm and muscle cells

**Figure 2:**
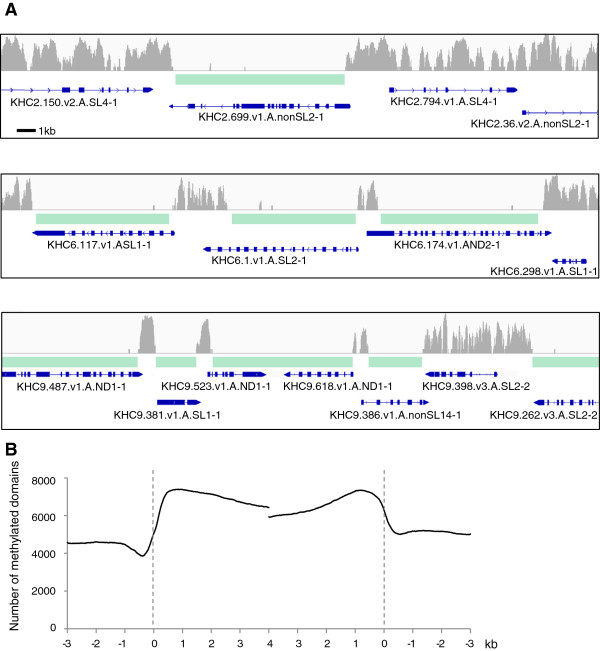
**Position of methylated domains in the sperm genome. (A)** Examples of typical CXXC-affinity purification followed by deep sequencing (CAP-seq) profiles of 30 kb. Read densities (gray) are as follows: KHC2: 2,755,136 to 2,785,136 (top), KHC6: 1,048,981 to 1,078,981 (middle) and KHC9: 4,508,000 to 4,538,000 (bottom). Genes (KH2010) are annotated below the CAP-seq profiles in blue. Methylated domains are indicated by green bars. **(B)** Methylated genes in sperm (genes with methylated domains with more than 80% overlap) were aligned at the 5′ end (left dashed line) or the 3′ end (right dashed line), and the total number of methylated domains are plotted.

The marked difference in global 5mC levels observed between adult tissues could partly reflect differences in locus-specific patterns of gene body methylation. To investigate this possibility genomewide, we chose sperm and adult muscle cells as representative tissues with high and low levels of global 5mC. Previous investigation of a total of 100 kb of randomly selected genomic regions by BS-seq showed that sperm DNA consists of domains with extreme methylation levels: either almost 100% methylated or 0% methylated. The boundaries between the methylated and unmethylated domains are sharp [11]. We therefore subjected sperm DNA to CAP-seq, which specifically enriches the unmethylated fraction of DNA via its affinity for the CXXC domain of mouse methyl-CpG-binding domain protein 1 (Mbd1) [26], to determine the unmethylated domains in the genome. After an initial optimization of salt-wash conditions (see Methods and Additional file 1: Figure S2), CXXC affinity purification of *C. intestinalis* unmethylated genomic fractions generated 28 million Illumina sequence reads, each mapped uniquely to the reference *C. intestinalis* KyotoHoya (KH) genome assembly. The CXXC-enriched regions appeared as stretches of DNA sequences corresponding to unmethylated domains (Figure 2A). As a control, reads derived from unfractionated input DNA covered most of the genomic region excepting repetitive regions. We deduced methylated domains as regions not covered by CXXC reads. The mapping results are in accord with our previous bisulfite data [11] (Additional file 1: Figure S3).

The resulting DNA methylation map clearly reflects a genomewide mosaic methylation pattern comprising methylated and unmethylated domains. Methylated domains regularly colocalize with genes due to comprehensive gene body methylation (Figure 2A). As a result of the mapping, approximately 63.7 Mb of the genome were identified as methylated, of which 83.0% overlapped with annotated genes. Figure 2B shows the positional relationship between genes and methylated domains. The methylated domain is located centrally over a gene with depletion at the gene ends and a further dip just upstream of the transcription start site (TSS). General features of gene body methylation in *C. intestinalis* sperm are consistent with those in other organisms which harbor gene body methylation [13,14,17]. The boundary of each methylated domain is related neither to the first methionine nor to the termination codon (Additional file 1: Figure S4).

Occasionally, two genes are covered by one methylated domain when genes converge in opposite orientations. As shown in Figure 2A (bottom: *KH.C9.523.v1.A.ND1-1* and *KH.C9.618.v1.A.ND1-1*) and Additional file 1: Figure S5, tail-to-tail–oriented genes lack a 3′ drop in methylation, as the methylated domain continues from one gene body to the other. The transcriptional direction of these genes is evident because of the addition of 3′ poly(A) in expressed sequence tags (ESTs) (Ghost Database: *Ciona intestinalis* genomic and cDNA resources; http://ghost.zool.kyoto-u.ac.jp/cgi-bin/gb2/gbrowse/kh/). Their 5′ promoter and TSS are unmethylated. The genes concerned do not overlap, and no EST within the gap between them has been reported. It is unlikely that the intragenic region is too short to be detected as an unmethylated domain by CAP-seq, because the poly(A) sites of both genes are more than 500 bp apart, which is equivalent to numerous short unmethylated promoters that were successfully detected in this study (for example, in Figure 2A, bottom, between *KH.C9.381* and *KH.C9.523*, and between *KH.C9.618* and *KH.C9.386*). We found 157 similar tail-to-tail–oriented genes that were more than 500 bp apart and associated with a single, uninterrupted methylated domain.

**Figure 3:**
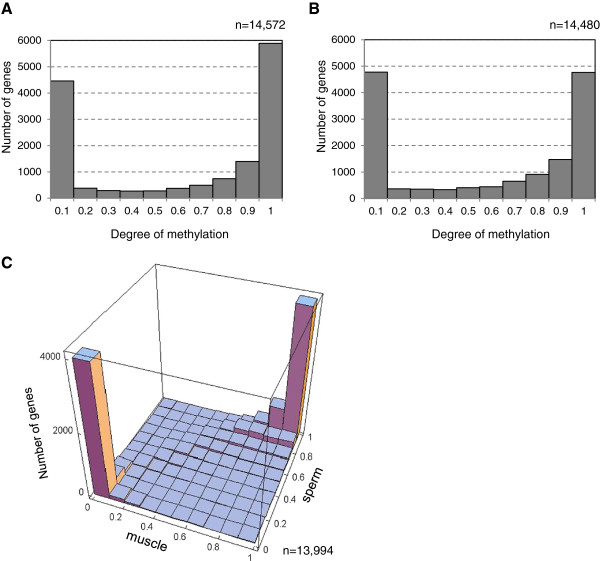
**Methylation degree of gene bodies in sperm and muscle. (A)** Frequency of genes with varying methylation degree in sperm. Methylation degree was represented by ratio of gene region overlapped with methylated domains. **(B)** Frequency of genes with varying methylation degree in muscle. Methylation degree was calculated as the proportion of methylated CpG sites in the gene. **(C)** Joint frequency distribution of the methylation degree of genes in sperm and muscle.

We then assessed the methylation status of each gene by selecting 14,572 reliable gene models corresponding to 95.4% of all annotated gene models, as the whole gene region is covered by input reads. The degree of methylation for each gene was calculated as a proportion of the transcribed region (from TSS to poly(A) site) that overlapped with the methylated domain. The frequency distribution of genes displayed a remarkably bimodal pattern in gene body methylation, corresponding to almost entirely methylated (>0.9) or almost entirely unmethylated (<0.1) states (Figure 3A). About 60% of the genes were methylated (>0.9) in sperm, which is in agreement with a previous estimate [11].

Next, we generated a list of methylated genes in muscle cells to compare with that of sperm. Because adult muscle DNA methylation appeared variable in frequency at each CpG site (Figure 1B), we mapped methylation levels for each CpG site using genomewide BS-seq data of *C. intestinalis* adult muscle cells [13]. This data set of 20.3 million reads mapped to unique genomic regions of the KH genome assembly and detected 1,200,307 methylated CpG sites (see Methods). The overall methylation statistics for the genome are equivalent to those in the original study. We found that 23.1% of the cytosine residues in the CpG context were methylated, whereas 21.6% were methylated in the study by Zemach *et al*. [13]. Non-CpG methylation was significantly lower than CpG methylation: 0.2% of cytosines in the CHG context and 0.4% in the CHH context were methylated, which are comparable to the data reported by Zemach *et al*. (0.3% and 0.3%, respectively) [13]. As shown in Figure 1B, the level of CpG methylation within methylated domains is lower in muscle cells (70.5% on average in the genomewide BS-seq data [13]) than in sperm cells (97.9% in a total of 100 kb of randomly selected genomic regions analyzed by BS-seq [11]). Next, we examined gene body methylation status in muscle for 14,480 genes, within which over 60% of CpG sites possessed ≥2 read coverage, corresponding to 94.8% of all gene models. Methylated cytosine residues in the genome were statistically defined (see Methods), and the degree of gene body methylation for each gene was represented by the ratio of methylated CpG sites to all CpG sites in the transcriptional unit. As in sperm, genes in muscle DNA were separated into highly methylated and entirely unmethylated categories (Figure 3B).

Are body-methylated genes identical in the two different cell types? Adult muscle and sperm cells arise from different cell lineages, and they express distinct gene sets [27]. We therefore expected to see some of these genes differentially methylated in sperm and muscle cells. Surprisingly, the methylation status in sperm and muscle for *C. intestinalis* genes was almost perfectly correlated (Figure 3C) (*r* = 0.915; *P* < 10^−6^ by 100,000 random permutations test). An independent statistical test also supported this result (Table 1). Very few genes showed differential methylation, such as methylated in muscle and unmethylated in sperm or vice versa (22 and 17 genes, respectively) (Table 1). Of these genes, 20 gene models were misannotated repeat sequences (Additional file 2: Table S1). This finding arose from a few methylated bisulfite clones that originated from genomic repeats that could not be distinguished by short-read sequencing [11]. We verified the remaining 19 nonrepetitive genes by BS-seq with gene-specific primers, 14 of which showed matching methylation status in sperm and muscle (Additional file 1: Figure S6). The remaining five genes proved refractory to bisulfite PCR but are unlikely to represent genes, as only one EST is reported from among a variety of tissues and stages.

**Table 1:** **Number of methylated and unmethylated genes in sperm and muscle**^**a**^

**Sperm**	**Muscle**		**Total**
	**Methylated**	**Unmethylated**	
Methylated	4,089	17	4,106
Unmethylated	22	3,998	4,020
Total	4,111	4,015	8,126

^a^Methylated and unmethylated genes were defined as genes with methylation levels less than 0.1 and greater than 0.9, respectively. We can reject the null hypothesis that the difference between the data sets occurred by random chance (*P* < 0.001 by Fisher’s exact test). Use of a different cutoff (methylated less than 0.05 and unmethylated greater than 0.95 or methylated less than 0.2 and unmethylated greater than 0.8) also rejected the null hypothesis (*P* < 0.001). A χ^2^ test also gave significant results (P < 0.001).

We found that the pattern of methylated and unmethylated genes is identical between muscle and sperm. This result implies that radical changes in gene body methylation status are not the major cause of decreased methylation levels in muscle cells. Instead, the reduced level of global 5mC in muscle is likely to be due to the lower average methylation level in methylated domains that are shared with sperm (see Additional file 1: Figure S3A).

### Stably or maternally expressed genes are methylated

**Figure 4:**
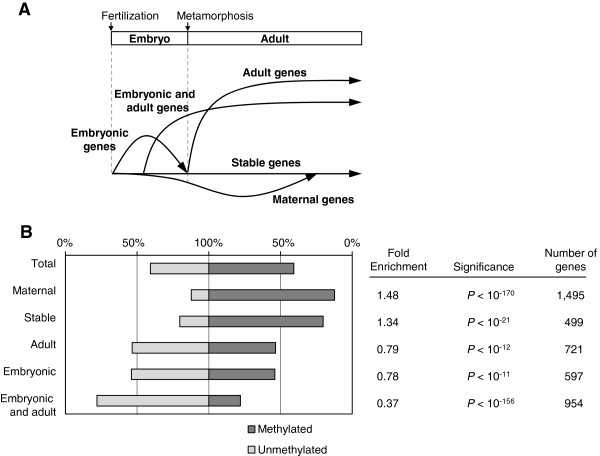
**Constitutively active genes are intensely methylated. (A)** A pattern diagram of gene expression in the life cycle of *Ciona intestinalis* modified from Azumi *et al*. [28]. Five gene groups were classified based on microarray analysis in the study. **(B)** The ratio of methylated and unmethylated genes for the five expression groups. Fold enrichment to methylated fraction, its significance (*P* values by Fisher’s exact test with Bonferroni correction) and number of genes examined are shown.

The data allowed us to compile a list of *C. intestinalis* genes in each tissue that are experimentally identified as methylated (<0.1) or unmethylated (>0.9) (Table 1). The strikingly well-correlated methylation status between sperm and muscle suggests that gene body methylation is not likely to be directly related to dynamic alteration of gene expression during tissue differentiation. Instead, the conservation of gene body methylation targets suggests that body-methylated genes may share features of expression across many cell types. To test this hypothesis, we investigated the association between methylation status and gene expression profiles during the life cycle. Expression microarray analysis of multiple developmental stages grouped genes into five classes: maternally expressed, stably expressed, embryonic, embryo plus adult, and adult [28] (Figure 4A). We found that stably expressed and maternally expressed genes were strongly overrepresented among methylated genes, whereas embryonic, embryo plus adult, and adult genes were preferentially unmethylated (Figure 4B). We also assessed the methylation status of regulatory genes which are zygotic controllers of *Ciona* embryogenesis [29]. Of 103 genes, 2 were methylated, 94 were unmethylated and 7 were intermediate (Additional file 2: Table S2). Interestingly the two methylated genes, *ets/pointed2* and *LAG1-like5*, are expressed ubiquitously during multiple developmental stages (UniGene Cin.23802 [UGID:3499610] and UniGene Cin.28377 [UGID:3504185], respectively). The results did not change significantly when different methylation thresholds were applied, that is, methylated less than 0.05 and unmethylated greater than 0.95 or methylated less than 0.2 and unmethylated greater than 0.8.

It has been proposed that DNA methylation in gene body regions regulates splicing [21,30,31]. We therefore investigated the relationship between gene methylation status and number of transcriptional variants. About 30% of all gene models were annotated with different transcriptional isoforms (*C. intestinalis* KH gene models), but we found no significant bias of these genes between the methylated and unmethylated groups (Additional file 2: Table S3).

### Role of promoters in establishing gene body methylation pattern

**Figure 5:**
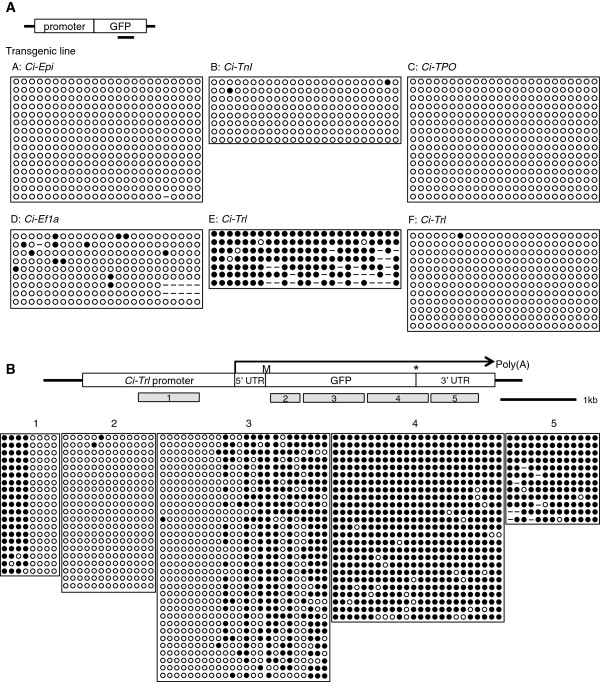
**DNA methylation analysis of transgenes driven by *C. intestinalis *gene promoters. (A)** Bisulfite sequencing of transgenic green fluorescent protein (GFP) gene bodies driven by *Ciona* promoters. Black bar represents the position of the analyzed area. Methylated CpG sites (solid), unmethylated CpG sites (open) and CpG sites that were mutated or failed to be sequenced (dashed line) are shown. Genomic DNA from larvae and juveniles was examined. **(B)** Same as **(A)**, but line E sperm DNA was examined by bisulfite sequencing. A region-spanning promoter, GFP and a 3′ untranslated region (UTR) in the transgene were analyzed by five primer pairs, displayed as 1, 2, 3, 4 and 5. A gene body methylation pattern with a boundary of unmethylated and methylated regions is established.

Our results support the view that gene body methylation is associated with genes that are active ubiquitously, but that profiles do not dynamically change according to levels of gene expression. This result led us to hypothesize that gene promoters have a role in determining DNA methylation patterns. To explore this idea, we used the *Ciona intestinalis* Transgenic Line Resources (see Methods), which holds transgenic lines expressing green fluorescent protein (GFP) under the control of various *C. intestinalis* promoters. We asked whether some of these lines acquired *de novo* gene body methylation in the transgene. The transgenic lines were established using the *Minos* transposon system [32-35] with promoter domains extending from 2 kb upstream of start codon, including an endogenous TSS and 5′ untranslated region, the enhanced GFP (eGFP) coding region followed by a Simian virus 40 (SV40) 3′ transcriptional termination sequence and a poly(A) signal. Transposons *per se* are not targeted by methylation in the *C. intestinalis* genome [24]. All transgenic lines that we investigated were GFP-positive, indicating that silencing of transgenes by DNA methylation, as frequently seen in plants and mammals, did not occur. In most of the transgenic lines, tissue-specific GFP expression was observed. BS-seq of GFP gene body region in lines A, B and C, which carry transgenes with promoters that are epidermis-specific (*Ci-Epi*), muscle-specific (*Ci-TnI*) and endostyle-specific (*Ci-TPO*), respectively, showed that almost all CpG sites are unmethylated (0% to 2.2% methylation) (Figure 5A). This finding seems to mirror the unmethylated status of endogenous genes driven by these tissue-specific promoters (0% to 1.0%) (Additional file 1: Figure S7). Next, we looked at two transgenic lines, D and E, in which GFP is constantly expressed by a ubiquitous class of promoters. Line D, whose promoter originated from the body-methylated *Ci-Ef1a* gene (96.4%) (Additional file 1: Figure S7), displayed low-level methylation in GFP (6.3%) (Figure 5A). Line E, also including the promoter of the endogenously body-methylated *Ci-Trl* gene (88.5%) (Additional file 1: Figure S7), acquired intensive DNA methylation in the GFP gene body (97.2%) (Figure 5A). To determine whether the gene body methylation status in the transgene of line E is determined by the *Ci-Trl* promoter, we examined a parallel transgenic line, line F. Line F has the same transgene as line E, but it is inserted at a different genomic locus by random integration (genomic position KHC2:275935). In this case, however, gene body methylation was absent (0.3% methylated) (Figure 5A). We conclude that, contrary to the hypothesis under examination, the promoter itself did not consistently trigger gene body methylation downstream.

To investigate why line E obtained intense DNA methylation in the GFP gene body, we examined the methylation status of DNA surrounding the insertion site. The results showed that the transgene was inserted into genomic position KHC7:1904808, which is within the first intron of *polyglutamine-binding protein* gene (KH gene model: *KH.C7.662*). This is a ubiquitously expressed and gene body methylated gene. Transgene methylation in line E may therefore be due to a positional effect of the surrounding insertion site. Similar cases were seen in transposons in *C. intestinalis*[24]. Repetitive copies inserted in introns of methylated genes were passively methylated as a part of the methylated domain, whereas other copies located in an unmethylated area were methylation-free. Although these findings do not account for the domain organization of DNA methylation, they emphasize that promoter identity is unlikely to be the primary determinant of gene body methylation status.

Interestingly, although DNA methylation in line E is specifically targeted to the gene body, an unmethylated 5′ domain was detected (Figure 5B). This unmethylated 5′ area across the TSS of the transgene expands from about 800 bp upstream to 1 kb downstream of the TSS, including an exogenous eGFP sequence. The transgene is expressed from the intron of methylated host gene, owing to the unmethylated promoter. Therefore, this transgene recapitulates promoter hypomethylation in spite of dense methylation downstream. Given that endogenous promoters are incompatible with DNA methylation, they are likely to be important components in shaping the 5′ edge of the methylated gene body.

## Discussion

Whereas gene body methylation is the most widely conserved DNA methylation pattern in eukaryotes, its discovery is rather recent. To explore its mechanism and biological significance, genomewide DNA methylation has been investigated in various animals by BS-seq, but so far comparison between different tissues or cell types within a specific species has not been reported. In this first methylome comparison between *C. intestinalis* sperm and muscle, we found that the targets of gene body methylation are identical in these highly dissimilar cells. During spermatogenesis, sperm-specific gene expression alters as cells mature [36]. Although most genes are silenced when canonical histones are exchanged with protamines, a small number of genes whose expression is essential for sperm cells continue to be expressed. Considering the dynamic alteration of gene expression, the DNA methylation pattern we observed in the sperm genome, which is identical to that in muscle cells, does not reflect the gene expression status of a specific spermatogenesis stage. Given that the paternal genome of *C. intestinalis* does not undergo genomewide demethylation and reestablishment of methylation pattern after fertilization, the methylation pattern in sperm may be a default epigenetic state.

A constant DNA methylation pattern was also reported in the sea urchin, where methylated and unmethylated genome fractions in sperm, embryos and adult intestines did not appear to exchange, as measured by reassociation kinetics between these genomes [37]. In the current study, we employed higher-resolution methods to investigate genomewide DNA methylation targets in single-gene resolution and found that methylated and unmethylated domains are constant throughout development. The idea that DNA methylation status dynamically changes to regulate gene expression during differentiation appears to be inapplicable to the deuterostome genomes, although the possibility that DNA methylation temporarily changes in specific genomic regions is not completely excluded. An additional possibility is that the distribution of 5-hydroxymethylcytosine (5hmC), generated by oxidation of 5mC by the ten eleven translocation (TET) family of enzymes, is dynamically regulated instead. However, it remains an open question at present when TET proteins and 5hmC appeared in evolutionary history.

About one-half of genes are methylated and the other half are unmethylated in *C. intestinalis*. We detected a clear enrichment of methylated genes in maternally and stably expressed genes (Figure 4B). Maternally expressed genes, comprising 35% of all genes, are largely housekeeping genes which encode proteins required for essential cellular functions [38]. Those mRNAs and proteins are stored in eggs in sufficient quantities to afford rapid consumption during early development. Later in development these genes maintain basal transcription ubiquitously, in a fashion similar to that of stably expressed genes, which are also methylated. The data therefore support the conclusion that the majority of methylated gene bodies are associated with ubiquitously expressed genes.

The molecular mechanism by which cytosine methylation is added preferentially to ubiquitously expressed genes is unclear. One of the conceivable scenarios was that a *cis* element embedded in the ubiquitous promoter solely controls gene body methylation; however, our transgenic analysis did not support this idea. It is conceivable that a specific combination of *cis* and *trans* elements in a promoter and gene coding region may be required for gene body methylation.

On the basis of our observations, the DNA methylation pattern may be determined by a combination of multiple mechanisms. At first, it seems that DNA methylation is predominantly targeted to the body of ubiquitous genes and leaves the 5′ promoter, intergenic region and tissue-specific genes unmodified. Instead, in the transgenic line E, the *Ci-Trl* promoter sequence integrated into the intron of endogenously methylated gene was primarily protected from DNA methylation. In addition, methylated intergenic regions of the subset of genes in a convergent orientation (Figure 2A) also suggested that DNA methylation is not always restricted to the gene body. The methylated intergenic regions may lack a property required to actively create the unmethylated promoter domain. One possibility is that trimethylation of lysine 4 of histone 3 (H3K4) deposited at an active promoter prevents methylation, as a mutually exclusive distribution of DNA methylation and H3K4 methylation has been reported in genomewide studies in mammals [39] and this histone modification is unable to bind *de novo DNMT*s [40].

## Conclusions

Herein we report that methylated and unmethylated gene groups are identical in *C. intestinalis* muscle and sperm cells, although their global levels of DNA methylation in the genome are different. The constant targeting of gene body methylation, regardless of cell type, indicates that DNA methylation is not a major regulator of tissue-specific gene expression. Instead, gene body methylation is linked to ubiquitously expressed genes, although this does not seem to be determined by their promoters alone. Overall, these studies indicate the presence of specific epigenetic states in ubiquitously expressed gene bodies.

## Methods

### Reference genome sequence and gene models

The *C. intestinalis* KH genome assembly and KH gene models (version 2010) were obtained from the *Ciona intestinalis* genomic and cDNA resources Ghost Database [41,42].

### Biological samples

*C. intestinalis* sperm DNA extracted from North Pacific specimens were kindly provided by Shota Chiba and William Smith, University of California, Santa Barbara, USA. Adult specimens were obtained from the Maizuru Fisheries Research Station of Kyoto University and the Misaki Marine Biological Station of University of Tokyo through the National BioResource Project of the MEXT, Japan. Tissues, eggs and sperm were obtained surgically. After fertilization, embryos were raised in filtered seawater at 18°C. Adult specimen and sperm of *C. intestinalis* transgenic lines were provided by *Ciona intestinalis* Transgenic Line Resources (http://marinebio.nbrp.jp/ciona/).

### Global DNA methylation analysis

Genomic DNA was prepared from embryos and adult tissues by the conventional phenol extraction method [43]. The DNA concentration was measured by using the Qubit fluorometer (Invitrogen, Carlsbad, CA, USA). Global DNA methylation level was measured using Methylamp Global DNA Methylation Quantification Kit (Epigentek, Farmingdale, NY, USA) according to the manufacturer’s instructions. Methylated DNA was used as a positive control. The analysis was repeated in triplicate.

### Bisulfite sequencing

Sodium bisulfite conversion of genomic DNA was conducted using BisulFlash DNA Modification Kit (Epigentek). Bisulfite PCR primers were designed using the MethPrimer tool and database (http://www.urogene.org/methprimer/index.html) (Additional file 2: Table S4). PCR products were cloned using the StrataClone PCR Cloning Kit (Agilent Technologies, Santa Clara, CA, USA), and randomly selected multiple clones were subsequently subjected to Sanger sequencing, aligned and analyzed for their methylation status.

### CXXC affinity purification and deep sequencing

Sperm DNA of North Pacific specimen was sonicated using a Bioruptor ultrasonicator device (Diagenode, Denville, NJ, USA) for 10 seconds on the high setting to produce fragmented DNA ranging from 200 to 800 bp with a peak of 500 bp. CAP-seq was performed as previously described with a minor change [26]. An initial optimization of the salt-wash condition was conducted ranging from 300 mM to 800 mM and adopted 400 mM. DNA (35 μg) was bound to the CXXC matrix in a 100 mM NaCl-containing column buffer, washed and then eluted using buffer containing 1 M NaCl. Eluted fractions were pooled, concentrated and precipitated. An Illumina library of affinity-purified and input DNA were prepared as previously described [26]. High-throughput sequencing was conducted using the Illumina Genome Analyzer IIx and HiSeq 2000 (Illumina, Inc, San Diego, CA, USA) with a read length of 50 bp.

### Analysis of high-throughput sequence data

Single end reads obtained through high-throughput sequencing were quality-filtered and mapped to the genome assembly using Bowtie with the –best option (http://bowtie-bio.sourceforge.net/manual.shtml). Mapped affinity-purified and input sequences in the form of BedGraph Track Format files (UCSC Genome Bioinformatics; http://genome.ucsc.edu/goldenPath/help/bedgraph.html) were processed and analyzed using Perl scripts [26]. Regions with sufficient read coverage were identified using H, L and G parameters, which are read height, length in base pairs and gap permitted in the length parameter, respectively. The parameters for CAP-seq were calibrated to identify hypomethylated domains in 40 kb of the cos41 genomic region previously investigated by BS-seq [11]. The parameters, H 4 L 90 G 700, were validated in irrelevant 60-kb regions in total, where BS-seq has been conducted before. The same parameters were used for the analysis of the input reads. Methylated regions in sperm were determined as regions with the input coverage and deprived of the affinity-purified coverage.

### Sperm methylation

The gene body methylation status of each gene was assessed by calculating a ratio of gene region overlapped with methylated domains using the intersectBed utility in BEDTools v2.11.2 [44].

### Muscle methylation

We retrieved BS-seq data of adult muscle [13] from the Sequence Read Archive database (http://www.ncbi.nlm.nih.gov/sra). Short reads were mapped to the KH genome assembly using Bismark v0.5.2 [45] with a default setting (http://www.bioinformatics.babraham.ac.uk/projects/bismark/). We discarded reads mapped to multiple genomic positions. Next, the coverage (*X*) and the number of converted short reads (*m*) for each cytosine residue was extracted from the mapping results by running the Bismark methylation_extractor. In the following step, we assessed the methylation status for every cytosine residue with *X* ≥ 2. We estimated an error rate that included the error of bisulfite conversion, sequencing and mapping. The error rate was calculated from the mapping result of a mitochondrial genome, which has no or very low DNA methylation [46]. The error rate was estimated to be 0.00091. We tested whether *m* is explained only by the estimated error rate, given *X* by binomial testing for each cytosine residue, by following the method described by Lister *et al*. [47]. Methylated cytosine residues were defined as those with calculated *P*-values <0.01.

### Microarray data analysis

Microarray data were derived from the study by Azumi *et al*. [28]. The corresponding KH gene model to the array probe sequence was searched by local BLAST with an E-value cutoff of 1E-20. Duplicated gene models were removed. Gene models which hit multiple probes belonging to different expression groups were retained.

### Transgene insertion sites

Transgene insertion sites in lines E and F were identified as described by Sasakura *et al*. [33].

### Accession number

The CAP-seq data have been deposited in the National Center for Biotechnology Information Sequence Read Archive under the accession number DRA000388-390.

## Abbreviations

5hmC: 5-hydroxymethylcytosine; 5mC: 5-methylcytosine; bp: base pair; BS-seq: genomewide bisulfite sequencing; CAP-seq: CXXC affinity purification and deep sequencing; DNMT: *DNA methyltransferase*; PCR: polymerase chain reaction.

## Competing interests

The authors declare that they have no competing interests.

## Authors’ contributions

MMS, AY and MO carried out the experimentation and data analysis. SS, ARWK and SW helped with the sequencing data analysis. ST performed the statistical analysis. YS provided the transgenic animals. MMS and AB conceived the study and wrote the article. AN coordinated the studies. All authors read and approved the final manuscript for publication.

## Supplementary Material

Additional file 1: Figure S1 RT-qPCR analysis of expression levels of *DNMT1*, *DNMT3I* and *DNMT3II* in adult tissues. Data were normalized to GAPDH expression. **Figure S2.** CXXC affinity purification efficiently enriched unmethylated fragments of *C. intestinalis* genomic DNA. Regions of *C. intestinalis* genome with various CpG frequency ranging from 0 to 38 unmethylated CpG sites in 500 bp were used to test the specificity of the CXXC affinity purification. The methylation status of 4 methylated domains (MDs) and 4 unmethylated domains (UMDs) were experimentally identified previously [11]. **Figure S3.** CAP-seq results were validated by bisulfite sequencing data. **Figure S4.** The boundary of each methylated domain is not related to the first methionine nor termination codon. Methylated genes in sperm were aligned at the first methionine (left dashed line) or termination codon (right dashed line). **Figure S5.** Tail to tail oriented genes lack a drop of methylation at their 3′ end. **Figure S6.** Validation of DNA methylation status of genes detected as differentially methylated in sperm and muscle cells. **Figure S7.** DNA methylation analysis of endogenous genes whose promoters used to drive transgenic GFP. Click here for file

Additional file 2: Table S1 Differentially methylated gene models and their verification. **Table S2.** Methylation status of embryonic regulatory genes. **Table S3.** Numbers of genes with or without transcriptional variants in methylated and unmethylated gene groups. Methylated and unmethylated genes were defined as genes with methylation level < 0.1 and >0.9, respectively. We cannot reject the null hypothesis (*P* = 0.31 by Fisher’s exact test). **Table S4.** Primers used in this study. Primers for bisulfite sequencing and RT-qPCR are listed. Click here for file
