# Supplementary material for: Identical sets of methylated and nonmethylated genes in Ciona intestinalis sperm and muscle cells
Source: Epigenetics Chromatin. 2013 Nov 11;6:38. doi: 10.1186/1756-8935-6-38 (PMC3827831; doi:10.1186/1756-8935-6-38)
Supplement: Additional file 1: Figure S1 — RT-qPCR analysis of expression levels of DNMT1, DNMT3I and DNMT3II in adult tissues. Data were normalized to GAPDH expression. Figure S2. CXXC affinity purification efficiently enriched unmethylated fragments of C. intestinalis genomic DNA. Regions of C. intestinalis genome with various CpG frequency ranging from 0 to 38 unmethylated CpG sites in 500 bp were used to test the specificity of the CXXC affinity purification. The methylation status of 4 methylated domains (MDs) and 4 unmethylated domains (UMDs) were experimentally identified previously [11]. Figure S3. CAP-seq results were validated by bisulfite sequencing data. Figure S4. The boundary of each methylated domain is not related to the first methionine nor termination codon. Methylated genes in sperm were aligned at the first methionine (left dashed line) or termination codon (right dashed line). Figure S5. Tail to tail oriented genes lack a drop of methylation at their 3′ end. Figure S6. Validation of DNA methylation status of genes detected as differentially methylated in sperm and muscle cells. Figure S7. DNA methylation analysis of endogenous genes whose promoters used to drive transgenic GFP. [file 1756-8935-6-38-S1.pdf]

Figure S1.

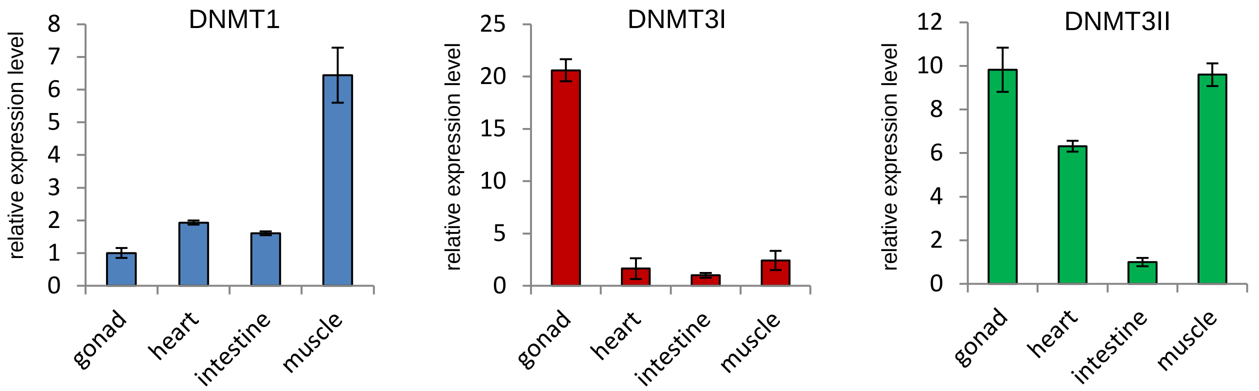

DNMT1
relative expression level
0
1
2
3
4
5
6
7
8
gonad
heart
intestine
muscle
DNMT3I
relative expression level
0
5
10
15
20
25
gonad
heart
intestine
muscle
DNMT3II
relative expression level
0
2
4
6
8
10
12
gonad
heart
intestine
muscle

Figure S2.

locus	Number of CpGs	Number of unmethylated CPGs	Input / affinity purified
MD2	8	0	
MD4	13	0	
MD1	18	0	
MD3	22	0	
UMD6	13	13	
UMD4	21	21	
UMD7	32	32	
UMD3	38	38	

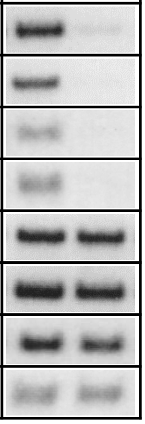

Figure S3.

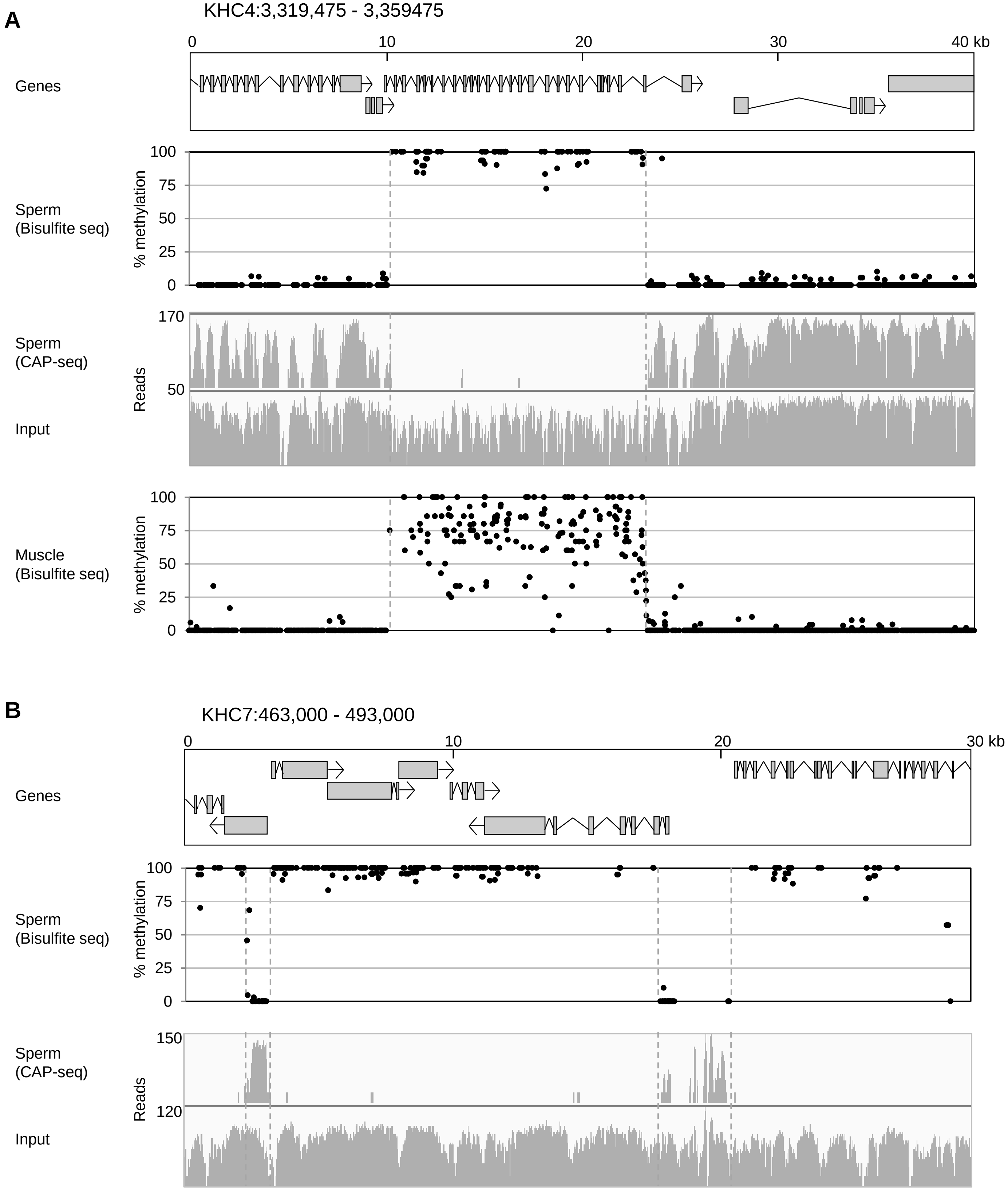
A
KHC4:3,319,475 - 3,359475
0
10
20
30
40 kb
Genes
Sperm
(Bisulfite seq)
% methylation
100
75
50
25
0
Sperm
(CAP-seq)
170
50
Reads
Input
Muscle
(Bisulfite seq)
% methylation
100
75
50
25
0
B
KHC7:463,000 - 493,000
0
10
20
30 kb
Genes
Sperm
(Bisulfite seq)
% methylation
100
75
50
25
0
Sperm
(CAP-seq)
150
120
Reads
Input

Figure S3.

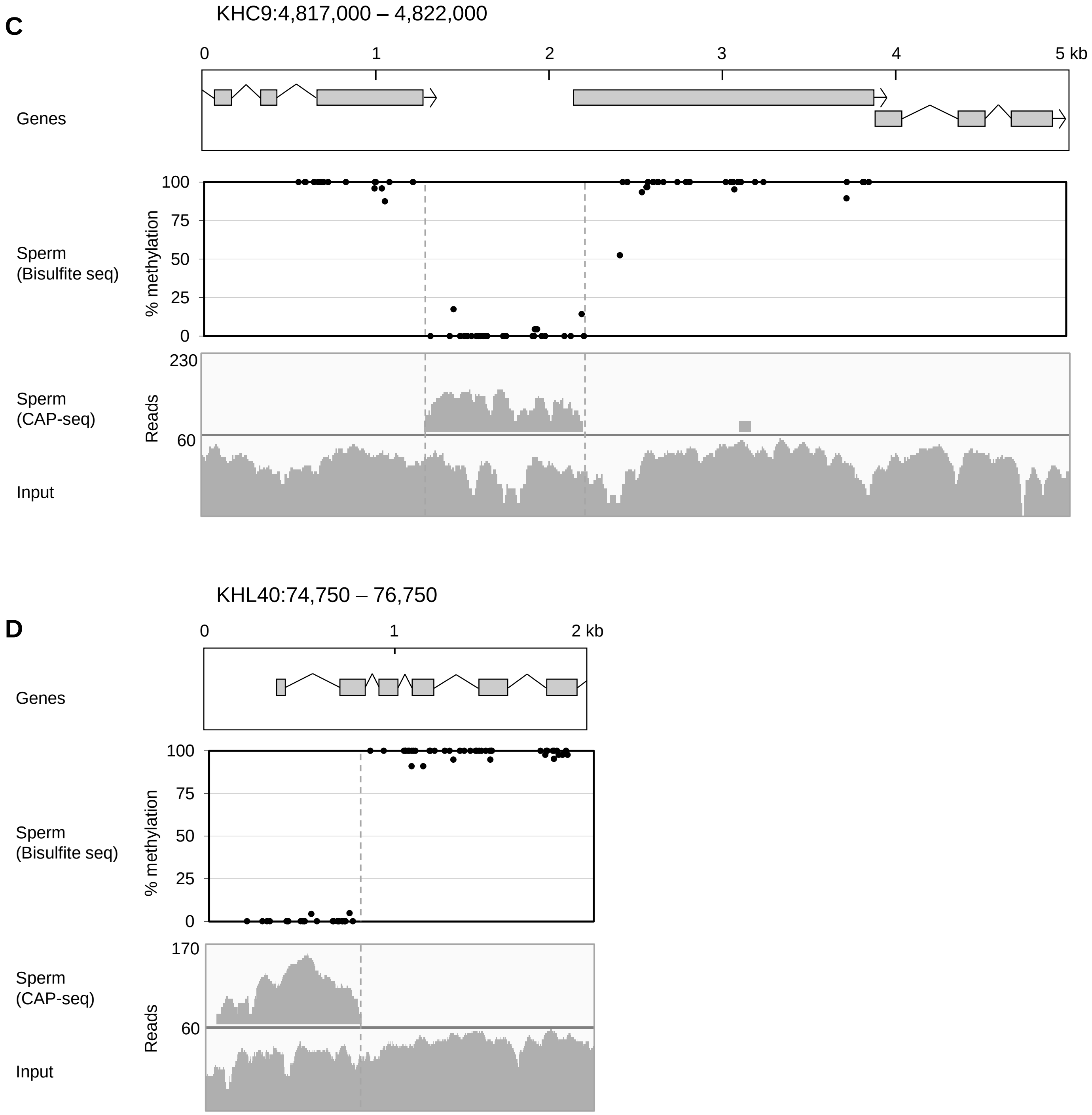
C
KHC9:4,817,000 – 4,822,000
0
1
2
3
4
5 kb
Genes
Sperm
(Bisulfite seq)
% methylation
100
75
50
25
0
Sperm
(CAP-seq)
Reads
230
60
Input
D
KHL40:74,750 – 76,750
0
1
2 kb
Genes
Sperm
(Bisulfite seq)
% methylation
100
75
50
25
0
Sperm
(CAP-seq)
Reads
170
60
Input

Figure S4.

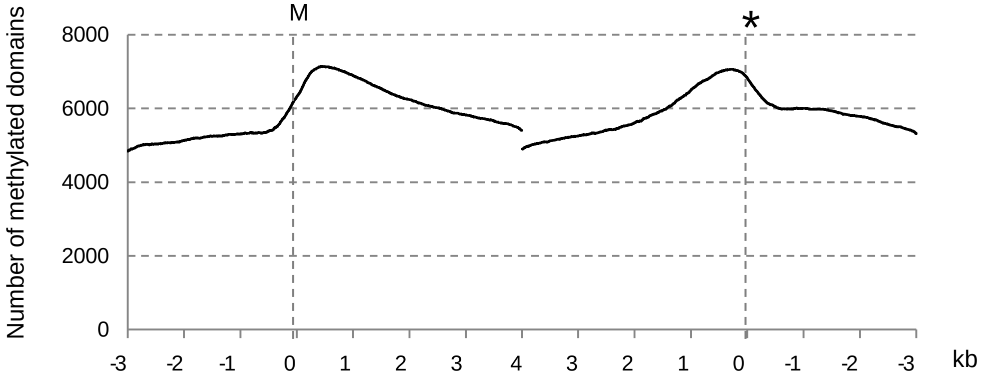

M
*
8000
6000
4000
2000
0
Number of methylated domains
-3
-2
-1
0
1
2
3
4
3
2
1
0
-1
-2
-3
kb

Figure S5.

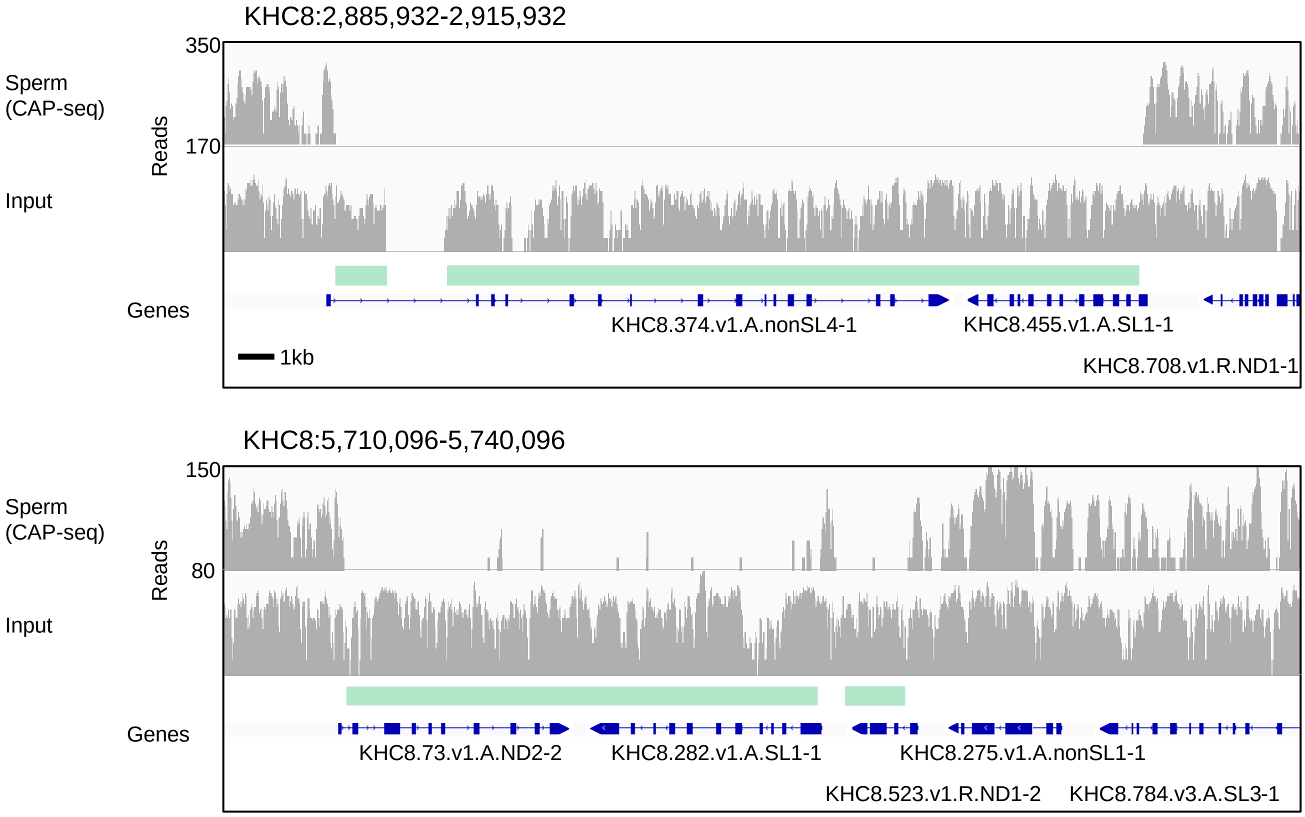
KHC8:2,885,932-2,915,932
350
170
Reads
Sperm
(CAP-seq)
Input
Genes
KHC8.374.v1.A.nonSL4-1
KHC8.455.v1.A.SL1-1
1kb
KHC8.708.v1.R.ND1-1
KHC8:5,710,096-5,740,096
150
80
Reads
Sperm
(CAP-seq)
Input
Genes
KHC8.73.v1.A.ND2-2
KHC8.282.v1.A.SL1-1
KHC8.275.v1.A.nonSL1-1
KHC8.523.v1.R.ND1-2
KHC8.784.v3.A.SL3-1

Figure S6.

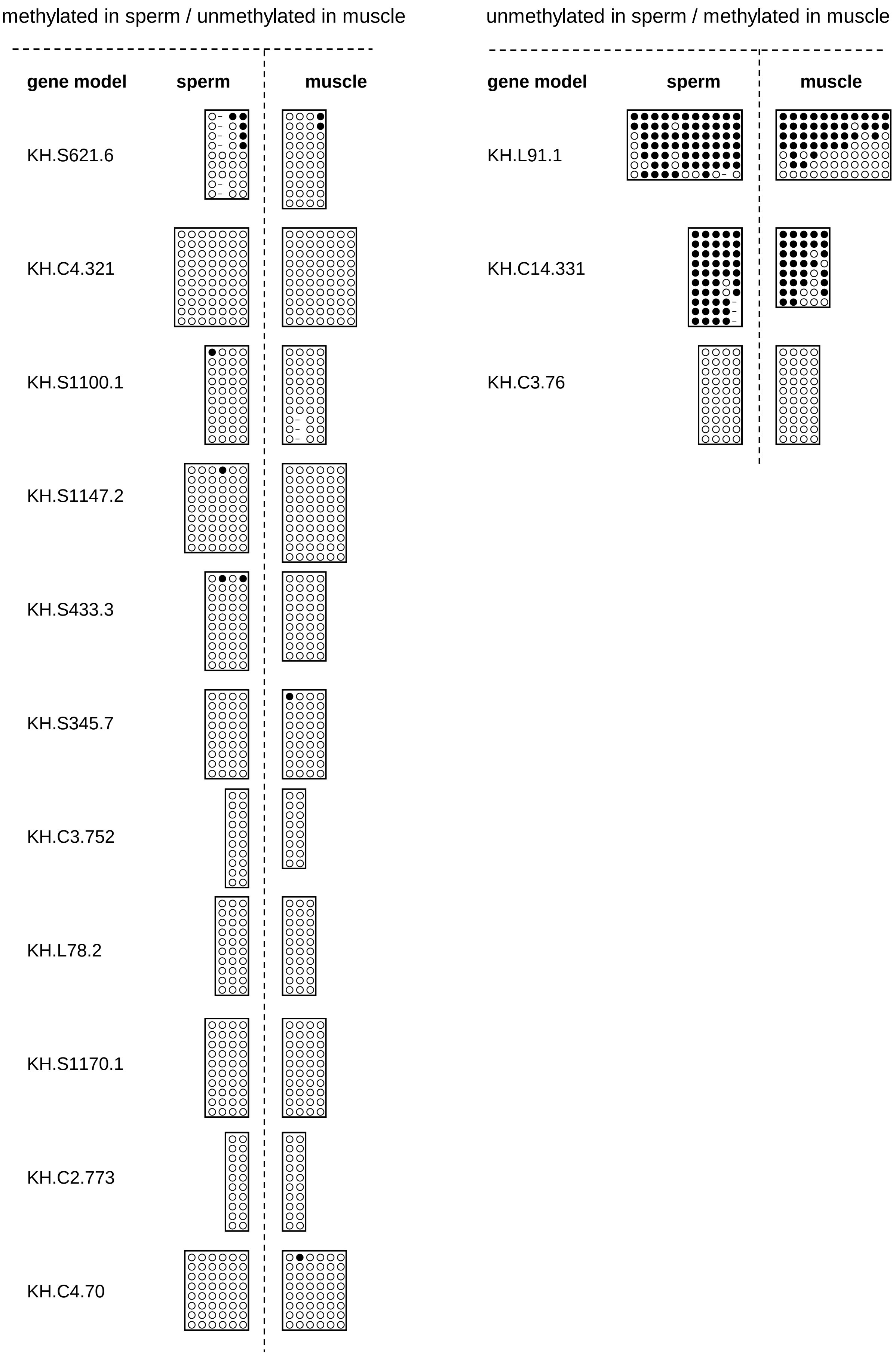
methylated in sperm / unmethylated in muscle
gene model
sperm
muscle
KH.S621.6
KH.C4.321
KH.S1100.1
KH.S1147.2
KH.S433.3
KH.S345.7
KH.C3.752
KH.L78.2
KH.S1170.1
KH.C2.773
KH.C4.70
unmethylated in sperm / methylated in muscle
gene model
sperm
muscle
KH.L91.1
KH.C14.331
KH.C3.76

Figure S7.

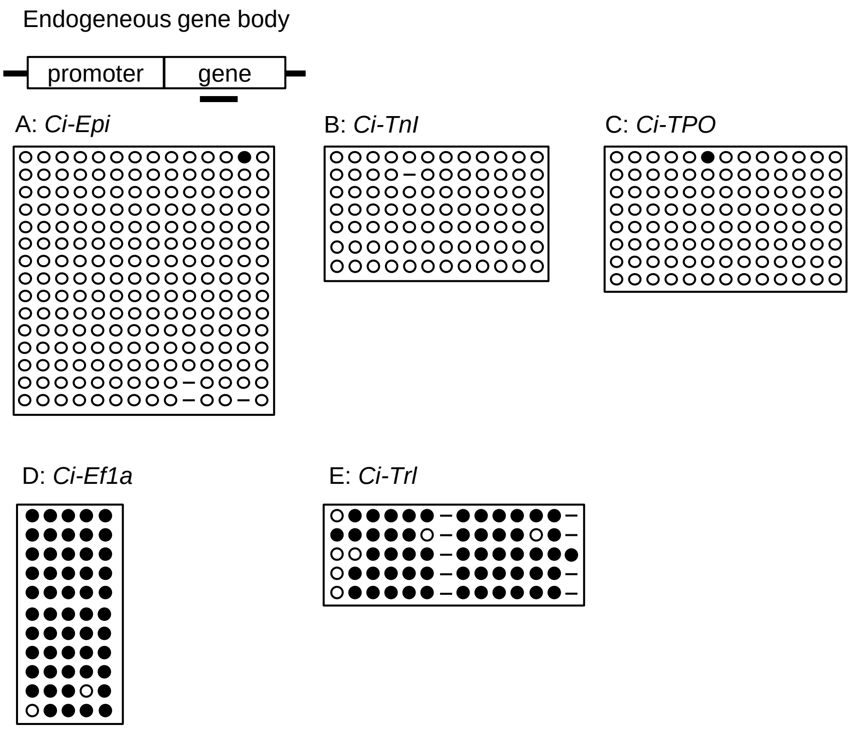
Endogeneous gene body
promoter
gene
A: *Ci-Epi*
B: *Ci-TnI*
C: *Ci-TPO*
D: *Ci-Ef1a*
E: *Ci-Trl*
