# Supplementary material for: Identical sets of methylated and nonmethylated genes in Ciona intestinalis sperm and muscle cells
Source: Epigenetics Chromatin. 2013 Nov 11;6:38. doi: 10.1186/1756-8935-6-38 (PMC3827831; doi:10.1186/1756-8935-6-38)
Supplement: Additional file 2: Table S1 — Differentially methylated gene models and their verification. Table S2. Methylation status of embryonic regulatory genes. Table S3. Numbers of genes with or without transcriptional variants in methylated and unmethylated gene groups. Methylated and unmethylated genes were defined as genes with methylation level < 0.1 and >0.9, respectively. We cannot reject the null hypothesis (P = 0.31 by Fisher’s exact test). Table S4. Primers used in this study. Primers for bisulfite sequencing and RT-qPCR are listed. [file 1756-8935-6-38-S2.pdf]

Table S1.

unmethylated in sperm / methylated in muscle

gene model	repetitive	bisulfite sequencing
KH.L76.9	yes	
KH.C7.531	yes	
KH.S870.1	yes	
KH.C3.546	yes	
KH.L18.68	yes	
KH.C2.416	yes	
KH.S1337.4	yes	
KH.L41.32	yes	
KH.L9.44	yes	
KH.C8.171	yes	
KH.S1352.1	yes	
KH.L20.63	yes	
KH.S1945.2	yes	
KH.S1225.1	yes	
KH.L91.1	no	conducted
KH.C14.331	no	conducted
KH.C8.666	yes	
KH.S931.2	yes	
KH.S1610.2	yes	
KH.S1777.1	yes	
KH.S2128.1	yes	
KH.C3.76	no	conducted

methylated in sperm / unmethylated in muscle

gene model	repetitive	bisulfite sequencing
KH.S621.6	no	conducted
KH.C4.321	no	conducted
KH.S494.6	yes	
KH.S1100.1	no	conducted
KH.S1147.2	no	conducted
KH.S433.3	no	conducted

KH.S345.7	no	conducted
KH.S345.5	no	failed to design specific primers
KH.C8.831	no	failed to design specific primers
KH.S1847.2	no	failed to design specific primers
KH.C3.752	no	conducted
KH.L78.2	no	conducted
KH.C4.100	no	failed to design specific primers
KH.S1170.1	no	conducted
KH.C2.773	no	conducted
KH.S610.3	no	failed to design specific primers
KH.C4.70	no	conducted

Table S2.

gene	KH	muscle methylation	sperm methylation	Methylated (M) / Unmethylated (U)
Achaete-Scute a like-2	L9.13	0.00	0.00	U
ADMP	C2.421	0.30	0.57	
AP-2-like2	C7.43	0.02	0.00	U
BMP2/4	C4.125	0.00	0.00	U
BMP3	C12.491	0.00	0.00	U
BMP5/7-like	C2.336	0.02	0.00	U
Brachyury	S1404.1	0.00	0.00	U
CAGF9	C3.330	0.11	0.22	
Cdx	C14.408	0.00	0.00	U
chordin	C6.145	0.40	0.67	
COE	L24.10	0.00	0.06	U
Delta-like	L155.7	0.42	0.56	
dickkopf	L20.29	0.00	0.00	U
Dll-B	C7.243	0.00	0.00	U
Dll-C	C7.770	0.00	0.00	U
DMRT1	S544.3	0.00	0.00	U
DUSP1.2.4.5	C1.10790	.00	0.00	U
E(spl)/hairy-a	C1.159	0.00	0.00	U
E(spl)/hairy-b	C3.312	0.00	0.00	U
E12/E47	C3.348	0.28	0.57	
ELK	C8.247	0.00	0.00	U
Emc	C7.692	0.00	0.00	U
Emx	L142.14	0.00	0.00	U
Eph1	C1.404	0.00	0.00	U
EphrinA-c	C3.52	0.00	0.00	U
EphrinA-d	C3.716	0.00	0.00	U
ets/pointed2	C10.113	0.97	0.98	M
FGF8/17/18	C5.5	0.00	0.00	U
FGF9/16/20	C2.125	0.00	0.00	U
Fli/Erg1	C4.539	0.00	0.00	U
Fli/Erg3	C10.148	0.00	0.00	U
Fos	C11.314	0.00	0.00	U

FoxA-a	C11.313	0.00	0.00	U
FoxB	C4.341	0.00	0.00	U
FoxC	L57.25	0.00	0.00	U
FoxF	C3.170	0.00	0.00	U
FoxH-a	C9.717	0.00	0.00	U
FoxH-b	L39.1	0.00	0.00	U
FoxP	C7.207	0.01	0.00	U
Fz4	C6.162	0.00	0.13	U
GATA-a	L20.1	0.00	0.13	U
GATA-b	S696.1	0.00	0.00	U
Hedgehog1	C8.359	0.00	0.00	U
Hex	L171.10	0.00	0.05	U
Hlx	C11.657	0.00	0.00	U
HNF4	C3.84	0.19	0.37	
Hox4	C1.959	0.00	0.10	U
Irx-B	C11.485	0.00	0.00	U
Irx-C	C3.67	0.00	0.00	U
Jun	C5.610	0.00	0.00	U
LAG1-like5	C3.732	0.99	1.00	M
lefty/antivin	C3.411	0.00	0.00	U
Lhx3	S215.4	0.00	0.10	U
Lmx	C9.616	0.00	0.00	U
Meis	C10.174	0.00	0.00	U
Mesp	C3.100	0.00	0.00	U
Mist	C3.308	0.00	0.00	U
Mnx	L128.12	0.00	0.00	U
Mox	C8.511	0.03	0.09	U
msxb	C2.957	0.00	0.00	U
MyoD	C14.307	0.00	0.00	U
MYTf	C1.274	0.00	0.00	U
Neurogenin	C6.129	0.00	0.00	U
NK4	C8.482	0.00	0.00	U
nodal	L106.16	0.00	0.00	U
noggin	C12.562	0.00	0.00	U
NoTrlc	C1.1116	0.00	0.00	U
Orphan Fox-2	C5.74	0.00	0.00	U

Orphan Wnt e	C6.25	0.00	0.00	U
Otp	C14.377	0.00	0.21	U
Otx	C4.84	0.00	0.11	U
Pax3/7	C10.150	0.00	0.00	U
Pax6	C9.68	0.00	0.00	U
PPAR	C8.143	0.23	0.35	
RAR	C9.580	0.03	0.14	U
ROR	C3.17	0.01	0.10	U
sFRP1/5	L171.5	0.00	0.11	U
sFRP3/4-b	C1.520	0.00	0.00	U
Six12	C3.553	0.01	0.31	U
Six36	C10.367	0.00	0.00	U
SMYD	S423.6	0.04	0.00	U
Snail	C3.751	0.00	0.00	U
SOCS1/2/3/CIS	L71.9	0.00	0.00	U
SoxB1	C1.99	0.00	0.00	U
SoxB2	S164.12	0.00	0.00	U
SoxC	C7.523	0.00	0.00	U
SoxF	C7.407	0.00	0.00	U
Tbx2/3	L96.87	0.00	0.00	U
Tbx6a	L8.11	0.00	0.00	U
Tbx6b/c/d	S654.3	0.00	0.00	U
TGFB not assigned1	C4.547	0.00	0.00	U
Tolloid	C12.156	0.00	0.03	U
TTF1	C10.338	0.00	0.13	U
Twist-like-1a/b	C5.416	0.00	0.00	U
Twist-like-2	C5.202	0.00	0.00	U
Wnt5	L152.45	0.00	0.00	U
ZF(C2H2)-2	C13.22	0.01	0.00	U
ZF(C2H2)-24	C5.154	0.00	0.00	U
ZF(C2H2)-25	C1.669	0.01	0.09	U
ZF(C2H2)-33	C1.777	0.00	0.00	U
ZF(C2H2)-34	C14.278	0.01	0.00	U
ZFC3H	C4.182	0.00	0.00	U
ZicL	S816.1	0.00	0.00	U

Table S3.

	Transcriptional variants		
	**single**	**multiple**	**Total**
**Methylated**	2,927	1,162	4,089
**Unmethylated**	2,822	1,176	3,998
**Total**	5,749	2,338	8,087

Table S4.

Primers for bisulfite sequencing

EF1a-F	GGGGTTTTTGTATGATATTTATTA
EF1a-R	AACTCCTCCAACATATTATCACC
Epi-F	AATTTAGTTTATTTTGAGAGTTTGA
Epi-R	AACAACAATATAATACAATTTCACA
Trl-F	ATTGGTTTTGGTAATTGTATTATTA
Trl-R	ACCTAATCTTTCTCCAACAATC
TnI-F	AAGTGTTATAGGTTTTTTTT
TnI-R	CAAATTCTTATTTTACTAAC
TPO-F	AGAGAGTAAAAGGTGTTTTATT
TPO-R	AATAAATAAACATCCAAACC
GFP_1-F	GTATTTATGGTGAGTAAGGG
GFP_1-R	AAACTTCAAAATCAACTTACC
GFP_2-F	GTTGATTTTGAAGTTTATTTGTATT
GFP_2-R	CAACTTATATCCCAAAATATTACC
GFP_3-F	GGTATTAAGGTGAATTTTAAGATT
GFP_3-R	CACCAAACCAACTAATAATAATAAC
GFP_4-F	GTTATTATTATTAGTTGGTTTGGTGTTAAA
GFP_4-R	AATACTATTACTTTATTTATAACCATTATA
KH.L91.1-F	GTAAGAGGATATTGGGATTT
KH.L91.1-R	AAACATTCCATTAAAACAAT
KH.C14.331-F	TTTTGTTATTGAAATGTGGG
KH.C14.331-R	CAATAAACAAATAACTTTATATTCT
KH.C3.76-F	TATAAAAGGGAAAAGGTTTT
KH.C3.76-R	TAAACACCACTAACCCATAA
KH.S621.6-F	ATAGTAGGTTATTTATTGTGATTAG
KH.S621.6-R	AACCTTAAATATTTTCTTAAATATA
KH.C4.321-F	ATTTGAAATTAGATGTGGAA
KH.C4.321-R	TCACCTCTAACTCTAACCAA
KH.S1100.1-F	ATGAATTGGATATATTTGAAGA
KH.S1100.1-R	ATTACATACAATATTTCACCCA
KH.S1147.2-F	TTTTTTGAGGAAGTTTTTTT
KH.S1147.2-R	TCATAACCTAATCCAAACAT

KH.S433.3-F	TATTAATTGAGTGGTTTTGT
KH.S433.3-R	AATCCAAAAAAATAAAATTC
KH.S345.7-F	GTAAGGGATATAATTGTTAGTT
KH.S345.7-R	TATTTATAAACAAAAAACATCA
KH.C3.752-F	TATTTAAGGTTGGGTGATTT
KH.C3.752-R	AACAACAATTTTATTTTCAA
KH.L78.2-F	AGGTTTATTTTTTTATATGG
KH.L78.2-R	TTAACTTCTTCACAAACTAA
KH.S1170.1-F	TTTTTTAGGTAAATTGTTGA
KH.S1170.1-R	ACCAAAAAAATAAATACCTC
KH.C2.773-F	ATTAATAGGGTTTTTTTTGA
KH.C2.773-R	AAACATTACAACACCTAAAA
KH.C4.70-F	GAAAGGTTGGTGTTAGTTTA
KH.C4.70-R	AAAAATCCAATTCATAAAAA

Primers for RT-qPCR

DNMT1-F	GATGGTAAGAAGCCCCGAAG
DNMT1-R	CCCGACATTTCATCGAACTT
DNMT3I-F	CGCAGCAAAAAGGTTATCGT
DNMT3I-R	TGGGGTTGGTAAGTAGATATTGC
DNMT3II-F	CAGATCAGGAAAAGCCCTCA
DNMT3II-R	TGTATAACCCCTTCGGCAAG
GAPDH-F	AAGGTTTTATGATAATTGTGTA
GAPDH-R	AAAATACTAACTTCTCCAAAC
